# Bilateral Fusion of the Semitendinosus and Long Head of the Biceps Femoris Muscles in a Male Cadaver

**DOI:** 10.7759/cureus.110270

**Published:** 2026-06-04

**Authors:** Sonia Asonganyi Eyong, Hayyan Chaudry, Luke Borgelt, Adetokunbo Elisha, Adel Maklad

**Affiliations:** 1 Department of Medical Education, The University of Toledo College of Medicine and Life Sciences, Toledo, USA

**Keywords:** anatomical variation, biceps femoris, cadaver study, fused muscle, hamstring, hamstring muscle fusion, semitendinosus

## Abstract

Bilateral variation of the semitendinosus (ST) and the long head of the biceps femoris (BFlh) muscles is an anatomically rare occurrence.

In this case report, we describe a male cadaver exhibiting bilateral fusion of the ST and BFlh muscles at their proximal origin on the ischial tuberosity, with the muscles separating distally into their respective muscle bellies. As far as we are aware, this bilateral fusion has not been previously documented in the literature; the only known report of fusion between the ST and BFlh muscles describes a unilateral fusion in a female cadaver, which was associated with a unilateral muscular slip that was not seen on either side of the bilateral fusion of our male cadaver. This bilateral fusion may have significant clinical implications regarding hamstring biomechanics, susceptibility to muscle strain, tendon injury, and possible sciatic nerve irritation or entrapment. Awareness of such variations may guide orthopedic surgeons, neurologists, radiologists, sports medicine specialists, and rehabilitation clinicians in accurate diagnosis and effective management of posterior thigh and lower extremity disorders.

## Introduction

The hamstring muscle group consists of the biceps femoris (BH; long and short heads), semitendinosus (ST), and semimembranosus muscles. The long head of the biceps femoris (BFlh), the ST, and the semimembranosus share a common proximal origin from the ischial tuberosity, whereas the short head of the BF arises from the linea aspera of the femur [1]. The hamstrings play a key role in knee flexion and hip extension, contributing significantly to gait, posture, and athletic movement [1,2].

Although the BFlh and the ST share a proximal aponeurotic connection, each muscle typically develops and functions as a distinct anatomical unit. The BFlh inserts onto the head of the fibula and the lateral tibial condyle. In contrast, the ST muscle inserts onto the upper anteromedial tibia as part of the pes anserinus, along with the sartorius and gracilis tendons [1,3]. The differing insertion sites allow these two muscles to contribute to both knee flexion and rotational control, with the ST assisting in medial tibial rotation and the BFlh assisting in lateral rotation.

Hamstring injuries commonly occur in athletic settings involving sprinting, kicking, and rapid acceleration or deceleration, largely due to the high mechanical loads during eccentric contraction of the muscles [4]. Rehabilitation for hamstring injuries often includes progressive loading, eccentric strengthening, and functional retraining [5].

While the hamstrings demonstrate some natural variability, proximal muscular fusion is extremely rare [6]. Current literature describes an isolated example of unilateral fusion between the ST and BFlh that was associated with a muscular slip [7] as well as occasional reports of accessory muscular slips or additional muscle heads without any fusion between the ST and BFlh [8]. The bilateral variation described in this case expands current understanding of anatomical variation of the hamstring and may provide clinical insight into hamstring injuries and rehabilitation.

Skeletal muscle forms from progenitor cells that are initially located in somites. These progenitor cells will leave the somite from the dermomyotome region and migrate into a given limb bud. Regulatory factors then allow for the embryological development of the skeletal muscle corresponding to the specific limb bud that these progenitor cells are located [9]. During embryological development, skeletal muscle located in the leg initially consists of fewer muscles than we see at the end of embryological development. However, the skeletal muscle that is initially present in the leg divides and forms more muscles. Division of the posterior thigh muscle into the BF (long head and short head), ST, and semimembranosus muscles occurs around the same time as the anterior thigh and lower leg. Additionally, evidence suggests that division of the anterior thigh muscle starts slightly prior to division of the posterior thigh muscle, while lower leg muscle division occurs later [10].

## Case presentation

The cadaver referenced in this study was obtained through a university anatomical donation program. Before his demise, the patient provided written informed consent for the utilization of his remains for medical education and research. After confirming the patient’s inclusion as a student cadaver, the body was drained of fluids and embalmed with a mixture of formaldehyde (35%), methanol (15%), phenol (5%), glycerin (10%), and water. The cadaver was then stored at 4 °C and occasionally moved to 0 °C to maintain preservation. Dissection and variation documentation were performed following ethical guidelines and university protocols.

**Figure 1:**
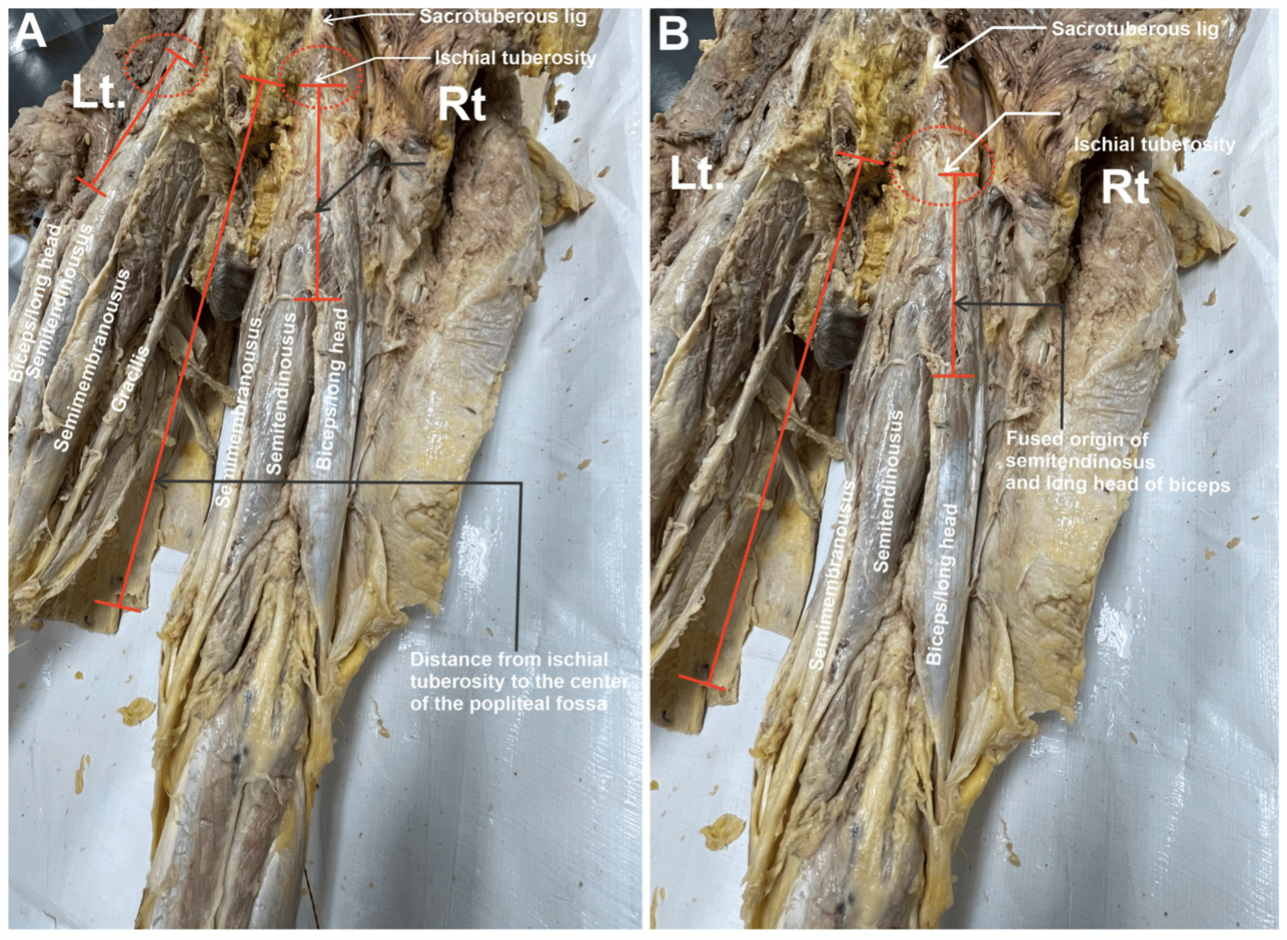
(A) Origination of the fused semitendinosus (ST) and long head of biceps femoris (BCFlh) muscles from the ischial tuberosity on the left and right thigh, respectively. The full length of the fusion of ST and BCFlh from the ischial tuberosity to the termination of fusion in the left thigh was 2 inches (5.08 cm) in length. On the right thigh, it was 2.5 inches (6.35 cm). (B) ST and BCFlh split into their respective muscle fibers.

**Figure 2:**
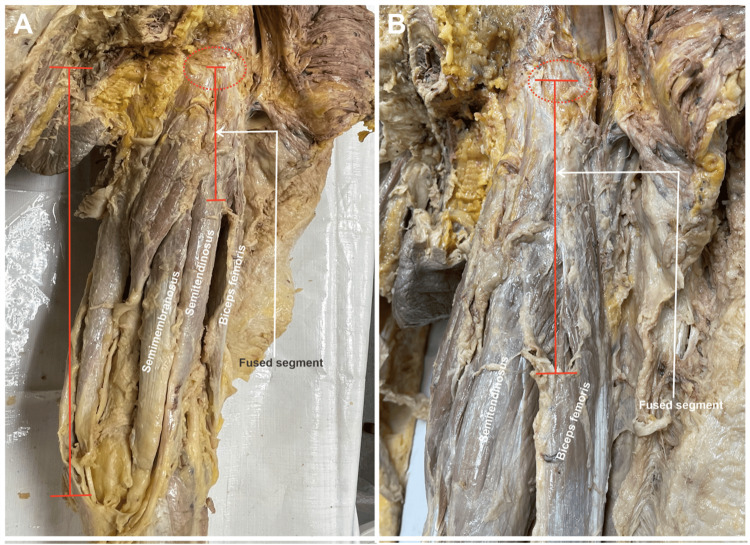
Fused segments with reference to the distance from the ischial tuberosity to the center of the popliteal fossa

**Figure 3:**
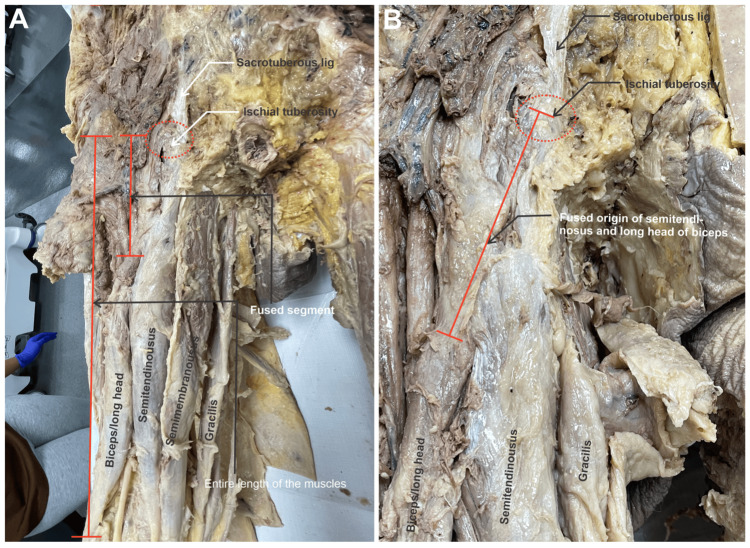
(A) Length of ST and BFlh muscles from ischial tuberosity; (B) Close-up view of fused origin ST: semitendinosus; BFlh: long head of biceps femoris

During dissection of the posterior compartment of the thigh in the anatomy lab at The University of Toledo College of Medicine, a rare variation of the hamstring muscles was discovered in a formaldehyde-fixed male cadaver. The cadaver was placed prone, and a midline incision was made from the sacrum to just superior to the anal canal. The skin and superficial fascia were reflected laterally to expose the gluteal and posterior thigh muscles. To visualize the ischial tuberosity, the gluteus maximus was reflected medially as seen in Figure 1 below. Then, attempted separation of the fascia between the hamstring muscles revealed bilateral fusion between the ST and the BFlh at the ischial tuberosity, their proximal origin, which can be seen in Figure 2. From the origin, the ST and the long head of the BFlh initially remained fused before splitting into separate muscle fibers, and this can be seen in Figure 3.

The fusion extended 5.08 cm (2 inches) on the left thigh and 6.35 cm (2.5 inches) on the right thigh. The width at the widest point was 3.81 cm (1.5 inches) on the left and 2.54 cm (1 inch) on the right.

This report in a male cadaver expands the limited literature, which previously described only a unilateral fusion in a female [7].

## Discussion

The anomalous fusions seen in this cadaver between the BFlh and ST may influence hamstring function. These fusions could change the range of motion or muscle torque during knee flexion and hip extension and may be more restrictive or more permissive. The ST, normally responsible for medial tibial rotation in semi-flexed knees [6], may have reduced rotational capacity. Additionally, as both muscles share tibial sciatic nerve innervation, fusion between the BFlh and ST may lead to nerve compression and posterior thigh pain. A previous report describes one case of a unilateral fusion between the BFlh and ST associated with a muscular slip. Additional reports describe muscular slips or accessory heads without fusion between the BFlh and ST [7,8]. Recognizing these variations enhances anatomical knowledge, aids in diagnosis of malformations, and informs treatment plans for hamstring injuries. It may also explain sciatic pain or reduced flexibility in affected patients.

Normal formation of the muscles of the posterior thigh requires proper embryological division of skeletal muscle of the posterior thigh [9]. Therefore, if this division is disrupted, some of the posterior muscles may remain a single undifferentiated muscle due to a lack of division of the precursor posterior thigh muscle. It is also possible that this division is not complete, meaning the BF (long head and short head), ST, and semimembranosus muscles all form, but some of the muscles are partially or completely fused with each other, such as the bilateral fusion of the ST and BFlh seen in this report. 

Bilateral fusion of the ST and BFlh is a rare anatomical finding that may have important functional implications. Normally, the hamstring muscles act as separate but coordinated units, allowing for efficient force generation and smooth movement across the hip and knee. When these muscles are fused, this independence may be reduced, which could limit normal muscle excursion and alter the force-length relationship, particularly during activities that require eccentric control such as running or deceleration. Although there is limited literature describing congenital fusion of the hamstrings, evidence from studies on post-injury fibrosis suggests that reduced tissue separation and increased stiffness can negatively affect muscle performance [11,12]. Previous work has shown that structural changes in the hamstrings following injury are associated with persistent alterations in contraction mechanics and joint loading patterns [13], while other studies report that increased muscle stiffness is linked to reduced flexibility and impaired functional movement [13]. In addition, injuries involving the intramuscular tendon, often associated with greater fibrotic change, have been shown to result in prolonged recovery and higher rates of reinjury [13]. When present bilaterally, these effects may contribute to symmetrical reductions in flexibility, strength, and endurance, and may increase reliance on compensatory movement patterns involving adjacent muscle groups. Taken together, these findings support the idea that structural fusion of hamstring components could have clinically relevant effects on mobility, function, and injury risk.

Fusion of the ST and BFlh may significantly alter the risk of injury in the region. Research suggests that non-modifiable variations in hamstring anatomy like the fusion in this report play a more significant role in injury than modifiable differences, such as the cross-sectional area of a muscle, which can be altered through exercise [5]. As hamstring muscles are commonly injured during sports, specific rehabilitation programs exist to aid in rehabilitation for hamstring muscles [6]. However, the effectiveness of these rehabilitation programs, which tend to focus on modifiable factors for injury recovery, can vary based on certain non-modifiable factors, including age and history of prior injury [6]. Incorporating an understanding of individual hamstring anatomy is one way in which hamstring muscle rehabilitation could be enhanced. As a result, it is important to recognize that anatomical variations, like in this case study, can affect how someone’s hamstrings function and how they recover if they suffer an injury.

Furthermore, the extensive fusion of the ST and BF can also present challenges in the diagnosis and management of severe injuries, particularly proximal hamstring avulsions. The biomechanics of a tear may cause a larger retraction of the muscle group, complicating surgical reattachment [14]. Additionally, variations in muscle bellies can mimic fibrotic scarring on magnetic resonance imaging or other anomalies such as soft tissue tumors [15]. Therefore, radiologists and surgeons need to be aware of this bilateral variant to prevent misdiagnosis and aid in preoperative planning for surgical procedures.

Limitations

The cadaver developed mold contamination before direct manual measurements could be performed; therefore, all measurements were obtained digitally, which may introduce minor variability. Although limited medical history was available for the cadaver, this remains a post-mortem case report, and the functional impact of the anatomical variation on the individual is unknown. Future studies examining similar anomalies in living subjects would be necessary to better understand their potential effects on mobility, function, and quality of life.

## Conclusions

Bilateral fusion of the ST and BFlh near the ischial tuberosity represents a rare anatomical variation. Awareness of such anomalies is important for clinicians, as they may affect hamstring function, lower extremity pain, and injury susceptibility.
